# Choosing wisely - when to mend a broken heart with ECMO?

**DOI:** 10.1186/cc13736

**Published:** 2014-02-20

**Authors:** R Scott Stephens, Eddy Fan

**Affiliations:** 1 Division of Pulmonary and Critical Care Medicine, Johns Hopkins University, Baltimore, MD 21205, USA; 2 Interdepartmental Division of Critical Care Medicine, University of Toronto, Toronto M5G 2 N2, Canada

## Abstract

Refractory cardiac shock in the cardiac surgical intensive care unit confers significant morbidity and mortality. Extracorporeal membrane oxygenation (ECMO) has become a common intervention for refractory cardiogenic shock when other therapies have failed. However, it is difficult to predict who will benefit from this costly, resource-intensive, but potentially life-saving technology. Here, we discuss the utility of a novel biomarker, serum butylcholinesterase, in determining survival in patients supported with ECMO following cardiac surgery.

## 

The specter of post-cardiotomy shock haunts cardiac surgeons and cardiac intensivists. Whether manifest as failure to septe from cardiopulmonary bypass, persistent low cardiac output with malperfusion, or sudden hemodynamic collapse in the cardiac surgical intensive care unit (CSICU), refractory cardiac shock confers significant morbidity and mortality. When inotropes, vasopressors, and intra-aortic balloon pumps fail, extracorporeal membrane oxygenation (ECMO) has become a common intervention for refractory cardiogenic shock. The work by Distelmaier and colleagues [1] continues important work aimed at determining who will benefit from this costly, resource-intensive, but potentially life-saving technology.

Approximately 1% of all adult patients undergoing cardiac surgery will experience post-cardiotomy shock [2]. ECMO has been used for post-cardiac surgical support since the early 1990s [3], but with increasing frequency as extracorporeal pumps, circuits, and oxygenators have improved. However, outcomes are still poor; only about 25% of patients supported with ECMO survive to hospital discharge [2]. Still, given the almost certain mortality of refractory cardiogenic shock, a subgroup of cardiac surgical patients will clearly benefit from ECMO; the difficulty lies in identifying this group of patients in a timely fashion.

Previous studies have attempted to identify predictors of successful weaning from ECMO, predictors of mortality on ECMO, and predictors of long-term outcomes after ECMO support [2,4-8]. The majority of these are retrospective, and more than a decade old, limiting their applicability to current ECMO technology. Factors associated with poor outcomes after ECMO for post-cardiotomy shock are not surprising: advanced age, complex operations, and pre-operative comorbidities are recurring themes. Distelmaier and colleagues should be congratulated for designing a prospective biomarker study to determine survival after ECMO.

Other groups have evaluated cardiac biomarkers as predictors of cardiac recovery during ECMO support with disappointing results [9]. Distelmaier and colleagues chose to examine levels of serum butyrylcholinesterase, which has been studied extensively in the context of neuromuscular blockade, but more recently has been reported to predict survival in cardiovascular and renal disease, albeit in relatively small populations [10-12]. Even after accounting for age, comorbidities, and duration of ECMO support, higher butyrylcholinesterase levels were associated with decreased mortality [1]. The mortality signal existed both in the short-term (30 days) and the long-term (up to 6 years). The vast majority of deaths occurred in the first year after ECMO implantation, highlighting that for those who survive the initial ECMO experience, long-term outcomes are reasonable. The patient population was modest (191 patients), but typical of a tertiary cardiac surgical practice, with isolated coronary artery bypass graft (CABG) surgeries, valve procedures, CABG-valve operations, and transplants represented.

The mechanism by which butyrylcholinesterase mediates the association with decreased mortality is not known. This tempers enthusiasm for this biomarker, as we neither understand the function of this enzyme, nor determinants of its level, nor the consequences of its deficiency. Indeed, in a study of stroke patients, cholinesterase activity was higher in patients than in matched controls [13]. Because butyrylcholinesterase levels were determined pre-operatively, it is not clear whether serum butyrylcholinesterase truly predicts ECMO-specific mortality, or whether it is simply a marker for elevated cardiac risk, and thus poor outcomes after cardiac surgery. Especially given the lack of an identified mechanism, changes in butyrylcholinesterase may simply be an epiphenomenon of critical illness. Accordingly, the utility of butyrylcholinesterase as a predictor of ECMO-specific mortality must be confirmed in future studies, and the biological mechanism elucidated. It is also unknown whether butyrylcholinesterase levels would be predictive of outcomes in other applications of ECMO, such as in acute respiratory failure.

If butyrylcholinesterase levels truly predict ECMO-specific outcomes, this biomarker could have significant implications. Compared to other uses of ECMO (for example, severe acute respiratory distress syndrome (ARDS)), the decision to undertake ECMO support in post-cardiotomy shock occurs in a compressed time frame. Whereas patients with severe ARDS typically decline to the point of requiring ECMO over a span of hours, post-cardiotomy cardiac failure can occur suddenly: an unanticipated failure to septe from cardiopulmonary bypass or an unheralded arrest in the CSICU. In the latter, there is little time to calculate a prognostic score such as the PRESERVE score proposed for ECMO and severe ARDS [14].

The ability to predict ECMO outcomes prior to surgery would arm surgeons and intensivists with more data to guide emergent decisions. Indeed, if this study’s findings are confirmed, one can envision development of a pre-operative 'game plan', with support options in the case of refractory shock explicitly laid out. While the pre-operative determination of appropriate support options for a given patient has some appeal, would preclusion of ECMO support based on a single pre-operative biomarker be acceptable to physicians and patients? Therapeutic decisions based on well-characterized biomarkers are common in other fields (for example, oncology) where mechanistic rationale and extensive clinical data exist. How strong must a biomarker, or indeed any prognostic data, be in order to justify withholding of ECMO support in an immediately life-threatening situation? These are important but unanswered questions. But as the cardiac surgical population becomes more complicated, ECMO technology improves, and mechanical circulatory support for refractory post-cardiotomy shock becomes more common, it will become critical to develop a rational framework for the use of this expensive and resource-intensive treatment. Distelmaier and colleagues have made an important step towards answering this question: not whether we could, but whether we should utilize ECMO to support a patient with post-cardiotomy shock.

## Abbreviations

ARDS: Acute respiratory distress syndrome; CABG: Coronary artery bypass graft; CSICU: Cardiac surgical intensive care unit; ECMO: Extracorporeal membrane oxygenation.

## Competing interests

The authors declare that they have no competing interests.
